# Decentralising IoT-Aware Business Processes Balancing Business Risk and Energy Consumption

**DOI:** 10.3390/s26206642

**Published:** 2026-10-20

**Authors:** Pedro Bandeiras Cardoso, Ana Respício, Dulce Domingos

**Affiliations:** LASIGE, Faculdade de Ciências, Universidade de Lisboa, 1749-016 Lisbon, Portugal; fc40338@alunos.fc.ul.pt (P.B.C.); alrespicio@ciencias.ulisboa.pt (A.R.)

**Keywords:** IoT, IoT-aware business processes, business process decentralisation, edge computing, cyber-physical systems, intelligent decision-support, business risk

## Abstract

As Internet of Things (IoT) devices become more capable, they participate in business processes (BPs), performing process activities and making control decisions that were traditionally assigned to central systems. This creates a design-time decentralisation problem: deciding where each process element should execute given limited device resources, communication failures, and battery constraints. Existing decentralisation approaches for IoT-aware BPs mainly focus on operational criteria, such as communication overhead, energy consumption, latency, or reliability. However, they provide limited support for analysing trade-offs between competing criteria and typically do not consider the business impact of failures. This paper proposes DECR, a decision-support methodology for decentralising IoT-aware BPs by jointly considering energy consumption and business risk. DECR models risk as expected business loss by combining reliability and business value, and formulates the decentralisation problem as a bi-objective generalised assignment problem, which is solved using an adapted bi-objective simulated annealing metaheuristic. Rather than a single solution, DECR provides process stakeholders with potentially Pareto-efficient alternatives to support the selection of decentralised models according to energy constraints and risk appetite, enabling business-aligned decisions. The methodology is illustrated through an irrigation case study based on a real-world IoT prototype and further assessed through a sensitivity analysis and a computational study.

## 1. Introduction

Advances in Internet of Things (IoT) technologies have enabled the integration of IoT devices into business processes (BPs), supporting the execution of context-aware workflows in distributed environments [1,2,3].

Through sensing and actuation capabilities, IoT-aware BPs can proactively adapt to their operational environment and adjust their behaviour accordingly [4,5]. In addition, as the capabilities of IoT devices increase, they become active participants in BPs, enabling local and autonomous decisions, including early aggregation and filtering of sensor data [6,7].

The applications of IoT-aware BPs are widespread across various sectors, including smart homes, healthcare, industry, smart cities, agriculture, transportation and logistics, and retail, where IoT devices contribute to the execution of critical BPs [8,9].

The use of IoT devices as active participants in BPs creates a design-time decision problem concerning where BP elements should execute. Assigning BP elements to IoT devices may reduce the number of exchanged messages and consequently increase the reliability of the overall BP, since communication tasks are major contributors to process failures. However, decentralising parts of a BP to IoT devices may increase overall energy consumption whenever energy-intensive activities are shifted to energy-constrained IoT devices, reducing the energy savings obtained from eliminating communication tasks. Therefore, decentralisation decisions involve conflicting criteria and require process stakeholders to analyse their trade-offs.

Although existing decentralisation approaches for IoT-aware BPs mainly focus on operational optimisation criteria, such as communication overhead, latency, response time, reliability, or energy consumption [10,11,12,13,14], they generally overlook the business consequences of failures, i.e., the risk they entail.

Moreover, most existing proposals either optimise a single criterion or aggregate multiple criteria into a single weighted objective [10,15,16], thereby limiting stakeholders’ ability to explore alternative decentralisation models that align with their operational constraints and risk acceptance criteria.

In contrast, DECR is conceived as a decision-support methodology in which decentralisation alternatives are evaluated simultaneously in terms of energy consumption and business risk, with risk representing the expected business loss associated with failures rather than reliability alone. More specifically, DECR presents the following contributions:

- The integration of reliability with business value to represent the business consequences of decentralisation decisions.
- The bi-objective generation of a set of potentially Pareto-efficient decentralisation alternatives rather than the prescription of a single solution. This aspect allows stakeholders to postpone the articulation of preferences (priorities, operational constraints, and risk acceptance criteria) until after the trade-off between energy consumption and business risk is made explicit.

The DECR methodology includes (1) A modelling approach for enriching IoT-aware BPs with the information required for decentralisation decisions. (2) Methods for computing energy consumption and risk considering the main BP control-flow pattern blocks and the execution probabilities of conditional flows [17]. The risk computation supports the definition of reliability and business value at different granularity levels (tasks and groups of tasks) [18]. (3) A formal bi-objective optimisation model for IoT-aware BP decentralisation, which is solved using an adapted bi-objective simulated annealing metaheuristic, thereby providing stakeholders with an approximation of the Pareto-efficient alternatives.

We showcase the application of DECR through a case study based on a real-world irrigation prototype specified with BPMN (Business Process Model and Notation). To enrich the BP model with the information required for decentralisation decisions, such as mutability of process elements, energy consumption, reliability, and business value, we define a BPMN extension. Furthermore, we assess DECR through a sensitivity analysis of the case study and a computational study using pseudo-randomly generated BP models.

The remainder of this paper is organised as follows. Section 2 analyses related work, while Section 3 presents the DECR methodology. Section 4 details the application of DECR to BPMN models, and, in Section 5, we discuss its application. Finally, Section 6 concludes the paper with a summary of our findings and future work.

## 2. Related Work

Decentralisation of IoT-aware business processes (BPs) involves a design-time decision problem in which stakeholders must determine where process elements should execute while balancing competing operational and business criteria. Existing approaches differ regarding the optimisation criteria they consider, the mechanisms used to generate decentralisation alternatives, and the degree of support provided for stakeholder decision-making.

In a centralised workflow approach, a single engine is responsible for process execution, orchestrating tasks by sending and receiving messages. In contrast, in a decentralised execution approach, the workflow itself is distributed among multiple locations. This requires partitioning the workflow and transmitting the partitions to the participants at each location. A partition groups together workflow elements, such as activities, gateways, control, and data flows [6,19,20,21]. In the literature, workflow partitioning is also known as fragmentation [19,22,23], decomposition [24,25], and modularisation [26].

A workflow can be fully or partially partitioned [20]. Full workflow partitioning approaches assign each activity to a different partition, maximising scalability, but often increasing communication overhead. In partial workflow partitioning approaches, a partition can have more than one workflow element, reducing the number of partitions and inter-partition interactions.

Early research on workflow decentralisation focuses on enhancing scalability and concurrency by distributing workflow partitions across multiple workflow engines. For example, within the Mentor project [27], workflows are distributed across processing entities, while preserving synchronisation and variable consistency.

For data-intensive scientific workflows, decentralisation proposals focus mainly on minimising data dependencies and data transfer volume and/or costs [20,28]. Additional constraints such as execution time [29,30], deadlines, resource capacities, and load balancing [31,32] are also considered.

With the advent of web services (WS), decentralisation approaches increasingly focus on reducing communication costs and improving the execution efficiency of distributed service compositions. Nanda et al. [10] formulate workflow partitioning as a variant of the automatic program parallelisation problem and generate partitions that minimise communication costs while maximising throughput of WS compositions specified with the Web Services Business Process Execution Language (WS-BPEL) [33]. Xue et al. [11,34] extend this work to support unstructured workflows through graph-based partitioning techniques, and Ai et al. [35] propose a genetic programming approach.

The use of cloud services has shifted the criteria for decentralisation to account for elastic and pay-per-use execution environments, optimising execution time, cost, resource usage, and load balancing [36,37,38,39,40,41,42,43]. In addition, when using a public cloud platform, privacy and confidentiality constraints have also been addressed [44,45,46]. More recently, proposals also consider reliability [47], energy consumption [14], or even both [16].

Within the IoT context, criteria for service composition in IoT include response time, cost, availability, scalability, reliability, security, energy, correctness, reachability, deadlock, safety, and liveness [48]. In IoT-aware BP decentralisation proposals, the most frequently considered criteria are communication overhead [12,49,50], reliability [13], energy consumption [16], latency, and response time [51].

In mobile and edge computing, partitioning and offloading methods share the same decision problem: selecting a set of alternative placements under multiple criteria and constraints, such as energy consumption and completion deadlines [52] or energy consumption and execution costs [53].

Beyond classical workflow operational criteria, related placement problems also introduce new sustainability criteria. For example, they consider the carbon footprint in microservice placement [54].

Although these works emphasise a broad spectrum of criteria, they mainly focus on operational and technical execution concerns such as communication overhead, latency, reliability, execution cost, and energy consumption. Existing approaches generally treat failures from a technical perspective, focusing on execution reliability, communication failures, or service availability. However, reliability alone does not capture the business impact of failures. Consequently, existing decentralisation approaches provide limited support for analysing business risk as the expected business loss resulting from failures.

Several decentralisation approaches account for multiple optimisation criteria simultaneously. For example, Fdhila et al. [15] optimise communication overhead together with Quality of Service (QoS) attributes, including execution cost, execution time, reputation, reliability, and availability, through weighted aggregation techniques. Ye et al. propose an algorithm that minimises energy consumption while meeting the predefined reliability requirements of IoT applications [16].

However, existing multi-criteria approaches commonly aggregate multiple criteria into a single weighted objective and provide only one final optimised solution. Consequently, stakeholders have limited support for analysing alternative decentralisation designs and their associated trade-offs. Weighted aggregation techniques require stakeholders to define trade-off preferences a priori and may hide relevant decentralisation alternatives that satisfy different operational constraints or risk preferences. From a decision-support perspective, decentralisation decisions should explicitly expose the trade-offs between competing criteria and provide stakeholders with alternative decentralisation models that can be evaluated according to operational constraints and organisational preferences.

To the best of our knowledge, no existing approach simultaneously: (i) models decentralisation considering risk by combining reliability and business value, (ii) formulates decentralisation as a bi-objective decision-support problem, and (iii) provides an approximation of the Pareto-efficient alternatives set to support stakeholder-driven selection among decentralised process models.

In contrast, DECR explicitly incorporates the business consequences of failures into decentralisation decisions by combining reliability with business value, and jointly considers this business risk and energy consumption as separate optimisation objectives to generate potentially Pareto-efficient alternatives for stakeholder decision support.

## 3. The Decision-Support DECR Methodology

This section presents DECR, a decision-support methodology for decentralising IoT-aware BPs at design time. DECR supports process stakeholders in deciding where mutable BP elements should be executed, considering the trade-off between energy consumption and business risk. Instead of providing a single optimal solution, DECR generates a set of potentially Pareto-efficient decentralisation alternatives, enabling stakeholders to select the BP model that best fits their energy constraints and risk acceptance criteria.

### 3.1. Decision Problem and Methodology Overview

The decentralisation of IoT-aware BPs can be formulated as a design-time decision problem. Given a BP model composed of fixed and mutable elements, stakeholders must decide which participant should execute each mutable element. Participants may include a central system, IoT devices, edge nodes, or other distributed execution environments.

Each assignment decision affects both the process’s energy consumption and its exposure to failures, and consequently, the related risk. In particular, moving computation closer to IoT devices may reduce communication overhead but also increase energy consumption or expose high-value business activities to less reliable execution environments. Therefore, decentralisation requires an explicit analysis of these two conflicting criteria: energy consumption and risk.

DECR supports this decision problem by generating alternative decentralised BP models. Each alternative corresponds to a possible assignment of mutable BP elements to the participants. Alternatives are evaluated according to two objective functions: energy consumption and business risk. The output of DECR is a set of non-dominated alternatives that approximates the Pareto frontier of the decision problem.

**Figure 1:**
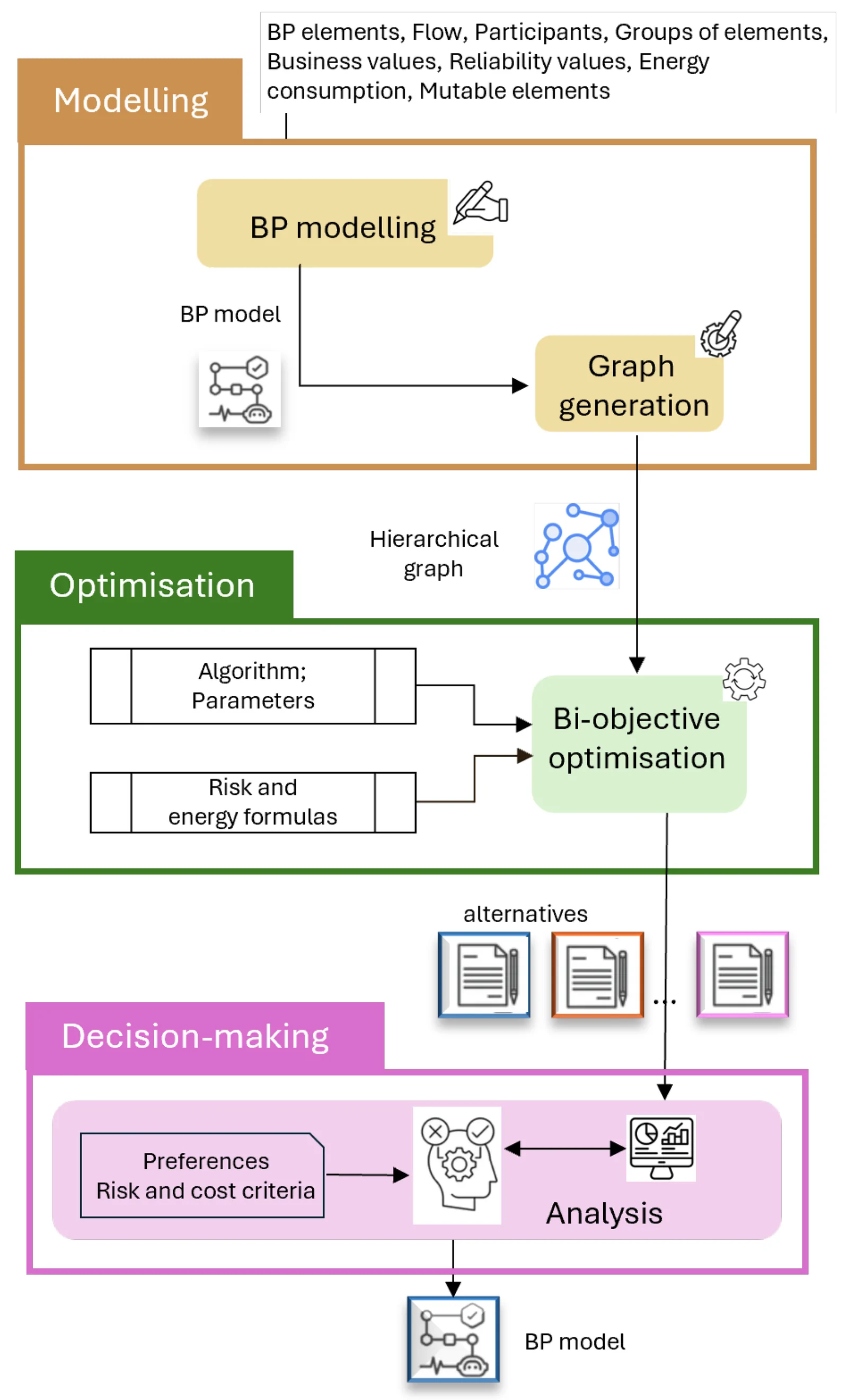
DECR methodology.

DECR comprises three layers, illustrated in Figure 1. In the modelling layer, the BP is modelled and enriched with the information required for decentralisation decisions. Then it is transformed into the hierarchical graph. In the optimisation layer, DECR searches for non-dominated alternatives; and in the decision-support layer, these alternatives are analysed by stakeholders according to their preferences, operational constraints, and risk acceptance criteria.

### 3.2. Language-Agnostic BP Graph Representation

DECR relies on a language-agnostic graph representation of the BP model. This representation abstracts from a specific modelling notation and captures the information required for decentralisation decisions: process structure, participants, mutability of process elements, transition probabilities, energy consumption, reliability, and business value. The graph serves as the input for the bi-objective optimisation procedure.

In this context, reliability is the counterpart of the failure probability; i.e., reliability is the probability of non-failure and equals 1 minus the probability of failure [17].

In practical applications, reliability values can be estimated from historical execution/failure data, device specifications, monitoring logs, or empirical measurements. Communication reliability can also be estimated from observed rates of message transmission and reception failures under relevant operating conditions. The business value represents the monetary impact on business objectives that might result from potential failures, considering their consequences [55]. It can be estimated, for instance, through a business impact analysis, expert judgement, or historical losses.

The hierarchical graph structure’s definition of subgraphs enables stakeholders to specify reliability and business values at different levels of granularity.

In this way, we formalise a BP model as a typed, directed, and weighted hierarchical graph G=(V,E,P,S,T,τ) [56], where:

- *V* is the set of nodes of *G*. A node represents an atomic BP element, such as a task, a gateway, or an event.
- *E* is the set of edges of *G*. A direct edge between two nodes can represent a control or a data dependency, distinguished by *T*.
- *P* is the set of participants that execute the BP elements represented as nodes.
- *S* is a set of subgraphs of *G*, including *G*. A subgraph $S_{s}=\left(V_{s},E_{s}\right)$, $S_{s}\in S$, $V_{s}\subseteq V$, and $E_{s}\subseteq E$, represents a group of BP elements. Subgraphs allow reliability, business value and energy consumption to be specified and computed at different levels of granularity. The function ρ defines the direct parenthood between two subgraphs and $\rho \left(S_{k}\right)=S_{s}$ iff $V_{k}\subset V_{s}$ and there is no $S_{r}$ with $V_{k}\subset V_{r}\subset V_{s}$.
- *T* defines the sets of types in *G*,
  $T=(T_{vs},T_{vj},P,T_{vfm},T_{ed},\left[0,1\right],R_{0}^{+},\left[0,1\right],R_{0}^{+})$,
  and τ is the corresponding typing function,
  $$\begin{array}{cc} \tau = & (\tau_{vs},\tau_{vj},\tau_{vp},\tau_{vfm},\tau_{ed},\tau_{ew},\tau_{energy},\tau_{energyOut},\tau_{energyIn},\\ \; & \tau_{rely},\tau_{relyOut},\tau_{relyIn},\tau_{bv}) \end{array}$$

The function τ is defined as follows:

- $\tau_{vs}:V\to T_{vs}$, where $T_{vs}=\{and$-split,xor-split,none}. A node with more than one outgoing edge is classified as an and-split or an xor-split node. An and-split node enables all its outgoing edges after completing its execution, while an xor-split node enables only one outgoing edge after completing its execution. The default value is none.
- $\tau_{vj}:V\to T_{vj}$, where $T_{vj}=\{and$-join,xor-join,none}. A node with more than one incoming edge is classified as an and-join or xor-join node. An and-join node starts its execution when all its incoming edges are enabled, and an xor-join node is executed as soon as *k* of the incoming edges are enabled, with *k* an integer and k≥1. The default value is none.
  These definitions of $T_{vs}$ and $T_{vj}$ support the representation of the five main BP design patterns: sequential, parallel, conditional, loop, and fault-tolerant [17].
- $\tau_{vp}:V\to P$, $\tau_{vp}\left(v\right)$ is the participant that executes *v*.
- $\tau_{vfm}:V\to T_{vfm}$, where $T_{vfm}=\{fixed,$mutable} and $\tau_{vfm}\left(v\right)$ defines whether *v* has a predefined execution participant or can be reassigned during decentralisation. Mutable nodes define the decision variables of the decentralisation problem.
- $\tau_{ed}:E\to T_{ed}$, where $T_{ed}=\left\{control,data\right\}$. This typing function distinguishes edges that represent control dependencies from edges that represent data dependencies.
- $\tau_{ew}:E\to \left[0,1\right]$, $\tau_{ew}\left(e\right)$ defines the probability that the control flow will pass through the edge *e*. The default value is 1.
- $\tau_{energy}:\left(V\cup S\right)\times P\to R_{0}^{+}$, $\tau_{energy}\left(i,j\right)$ defines the energy consumption of the execution of the element *i* by the participant *j*.
- $\tau_{energyOut}:\left(P\times P\right)\to R_{0}^{+}$, $\tau_{energyOut}\left(i,j\right)$ defines the energy consumption of sending a message from the participant *i* to the participant *j*.
- $\tau_{energyIn}:\left(P\times P\right)\to R_{0}^{+}$, $\tau_{energyIn}\left(i,j\right)$ defines the energy consumption of receiving a message by participant *j* from participant *i*.
- $\tau_{rely}:\left(V\cup S\right)\times P\to \left[0,1\right]$, $\tau_{rely}\left(i,j\right)$ defines the reliability of the execution of the element *i* by the participant *j*. Reliability can be defined at different levels of granularity.
- $\tau_{relyOut}:(P\times P\to \left[0,1\right]$, $\tau_{relyOut}\left(i,j\right)$ defines the reliability of sending a message from the participant *i* to the participant *j*.
- $\tau_{relyIn}:(P\times P\to \left[0,1\right]$, $\tau_{relyIn}\left(i,j\right)$ defines the reliability of receiving a message from the participant *i* to the participant *j*.
- $\tau_{bv}:\left(V\cup S\right)\to R_{0}^{+}$, $\tau_{bv}\left(i\right)$ defines the business value of the element *i*. The business value can also be defined at different levels of granularity.

### 3.3. Computation of Risk and Energy Consumption

Within DECR, a BP model is evaluated according to two decision criteria: business risk and energy consumption. DECR computes both criteria on the graph representation, using workflow-reduction rules for structured BP patterns [17]. It supports reduction rules for the five main pattern blocks, namely sequential, parallel, conditional, loop, and fault-tolerant. Therefore, the method only applies to graphs created from BPs structured with these five patterns. Likewise, subgraphs must follow the same structure.

The computation of risk values uses a quantitative methodology based on the concepts of reliability and business value, as defined in [18] and aligned with [57]. This risk formulation deliberately provides a quantitative and interpretable representation suitable for design-time comparison of decentralisation alternatives; it is not intended to represent every dimension of organisational risk. The risk value of a node $v_{i}$ executed by the participant *j* is calculated according to Equation (1).

(1) $$\begin{array}{cc} \; & Risk\left(v_{i}\right)=\left(1-\tau_{rely}\left(i,j\right)\right)*\tau_{bv}\left(i\right),where\;\tau_{vp}\left(v_{i}\right)=j \end{array}$$

For subgraphs, risk-related values may be defined directly or computed from their internal structure. If the business value of a subgraph is known, but its reliability is not, DECR first computes the subgraph reliability from the reliability of its constituent elements and then applies Equation (1). The same applies to business value if we only know the reliability. When neither reliability nor business value is known, DECR recursively computes the risk from the risk of the elements contained in the subgraph. This mechanism supports risk analysis at different granularity levels.

The formulae for these computations are shown in Table 1. To compute business value and risk, the formulae are grounded in the maximum function, measuring the impact of a failure as the maximum potential loss this failure entails. Hence, this approach quantifies the risk associated with a BP in the context of a worst-case scenario. The use of other functions, such as average and sum, does not adequately capture the essence of a worst-case scenario, as excessively low values may overshadow higher ones. This approach is consistent with risk assessment practices that focus on maximum potential loss rather than cumulative losses.

Other aggregation functions or richer risk models may be more appropriate when cumulative, correlated, time-dependent, or non-monetary consequences are relevant.

The reduction rules are applied until there is only one node. This method aligns with the hierarchical structure of the graph, where graph morphism functions ensure a direct one-to-one correspondence between subgraphs of level *l* and nodes of level l+1. The external edges (incoming and outgoing) of a subgraph of level *l* are represented as edges of the corresponding node of level l+1 (see Definition 3.2.1 of [56]).

In addition, we compute the energy consumption value by applying the Cardoso formulae [17], shown in Table 1.

### 3.4. Bi-Objective Assignment Model for Decentralisation Decisions

A solution for a graph G=(V,E,P,S,T,τ) representing a BP model is another graph $G^{\prime }=\left(V^{\prime },E^{\prime },P,S^{\prime },T,\tau^{\prime }\right)$ that represents the same model, with zero or more changes in the result $\tau_{vp}$ for mutable nodes.

The problem can be formulated as a bi-objective generalised assignment problem (GAP) without participants’ capacity constraints, as it involves assigning nodes to participants and evaluating the corresponding contribution to the objective functions. The current formulation assumes that all candidate assignments are feasible in terms of computational resources, without explicitly considering CPU, memory, storage, or other capacity constraints. In heterogeneous IoT environments, where devices have different resource capabilities, the current modelling extension should be enhanced to represent these differences, and the corresponding resource constraints should be considered when generating decentralisation alternatives. Meanwhile, nodes that cannot be executed by every participant due to resource limitations should be treated as fixed.

Let G=(V,E,P,S,T,τ) be the hierarchical graph representing the BP, where *V* is the set of process nodes, *E* is the set of edges, and *P* is the set of participants. In addition, let $M=\{v\in V:\tau_{vfm}\left(v\right)=\mathrm{mutable}\}$ denote the set of mutable nodes.

Define the binary decision variables:

(2) $$x_{vp}=\left\{\begin{array}{cc} 1, & \mathrm{if}\;\mathrm{node}\;v\;\mathrm{is}\;\mathrm{assigned}\;\mathrm{to}\;\mathrm{participant}\;p,\\ 0, & \mathrm{otherwise}, \end{array}\right.\;v\in V,\;p\in P.$$

Thus, only the variables associated with nodes in *M* constitute actual decentralisation decisions. For an assignment *x*, let G(x) denote the graph obtained by assigning each node according to *x*. Whenever adjacent nodes *u* and *v* are assigned to different participants, DECR introduces the corresponding send and receive operations. The energy consumption and reliability of these communication operations depend on the source and destination participants, exactly as specified by $\tau_{\mathrm{energyOut}}$, $\tau_{\mathrm{energyIn}}$, $\tau_{\mathrm{relyOut}}$, and $\tau_{\mathrm{relyIn}}$. The decentralisation problem can then be expressed compactly as

(3) $$\min_{x}\;\left(\mathrm{Energy}(G(x)),\mathrm{Risk}(G(x))\right)$$

subject to

(4) $$\sum_{p\in P}x_{vp}=1,\;\forall v\in V,$$

(5) $$x_{v,\tau_{vp}\left(v\right)}=1,\;\forall v\in V\setminus M,$$

(6) $$x_{vp}\in \left\{0,1\right\},\;\forall v\in V,\;p\in P.$$

Here, Energy(G(x)) and Risk(G(x)) are computed as detailed in Section 3.3. Constraints (4) force each node to be assigned to exactly one participant, while constraints (5) ensure that for every fixed node v∉M, its original participant is preserved. Finally, constraints (6) state that the variables are binary.

In the context of our optimisation problem, the *size* of a specific problem *SP*, denoted as Size(SP), is defined as the total number of possible assignments of participants to node execution. Consequently, the expression in Equation (7) defines Size(SP) for a problem with *k* mutable nodes and |P| participants.

(7) $$Size\left(SP\right)={\left|P\right|}^{k}$$

This exponential growth motivates the use of a metaheuristic algorithm to solve the problem.

### 3.5. Bi-Objective Simulated Annealing for Generating Decision Alternatives

To approximate the Pareto front of the decentralisation problem, DECR uses an adaptation of the bi-objective simulated annealing algorithm described in [58]. This established metaheuristic is used to explore feasible assignments and maintain an archive of non-dominated solutions identified during the search.

The algorithm explores the space of feasible assignments by iteratively modifying the participant assigned to one mutable node. During the search, it maintains an archive of non-dominated solutions, which represents the current approximation of the Pareto frontier.

In our bi-objective adaptation, we define two objective functions, fr and fe, which normalise, respectively, the risk and energy consumption to be minimised.

For a solution G(x), fr is normalised as follows:

(8) $$fr\left(G\left(x\right)\right)=\frac{Risk(G(x))}{Risk_{max}}$$

where $Risk_{max}$ is the risk of the process under the worst-case reliability configuration, in which for all nodes the reliability value is zero.

Similarly, for a solution G(x), fe is normalised as follows:

(9) $$fe\left(G\left(x\right)\right)=\frac{Energy(G(x))}{Energy_{max}}$$

where $Energy_{max}$ is obtained by considering the worst-case assignment for nodes (for each node *v*, we choose the participant *p* where we get the maximum value of $\tau_{energy}\left(v,p\right)$) and the worst case for communication links (for each pair of nodes v1 and v2, we choose the participants p1 and p2 where we get the maximum values of $\tau_{energyIn}\left(v1,p1\right)$ and $\tau_{energyOut}\left(v2,p2\right)$). The value for $Energy_{max}$ may not correspond to a feasible solution.

These normalisations place both objective values on comparable scales for use in the simulated annealing acceptance function.

To compare two solutions, the algorithm uses the dominance optimisation concept. For a bi-objective minimisation problem, a solution *a* dominates a solution *b* if both conditions are met:

1. *a* has no objective function value higher than the respective objective function value of *b*;
2. *a* has at least one objective function value lower than the respective objective function value of *b*.

If neither solution *a* nor *b* dominates the other, we assert that they are mutually non-dominated. Optimal solutions to a problem are those that are non-dominated.

Using these concepts, our optimisation algorithm maintains an archive of potentially non-dominated solutions. In this way, it archives a solution *s*′ as a potentially optimal solution only if *s*′ is non-dominated by any other solution in the archive.

The algorithm iteratively considers a set of potentially optimal solutions and starts by archiving the initial solution *s*, which corresponds to the graph representing the original BP model. The algorithm then generates a new neighbour solution *s*′ from *s*. Generating such a solution involves changing the participant responsible for executing a mutable node. For that, the algorithm randomly chooses a node $v_{k}$ such that $\tau_{vfm}\left(v_{k}\right)=mutable$, and randomly chooses a participant *p* with $p\neq \tau_{vp}\left(v_{k}\right)$. The algorithm then assigns *p* to $v_{k}$, making $\tau_{vp}\left(v_{k}\right)=p$, thus generating a new solution $s^{\prime }$, and proceeds by evaluating the objective functions for this new solution: $fr(s^{\prime })$ and $fe(s^{\prime })$.

To evaluate the objective functions, the graph is temporarily modified to accommodate communication flows between two adjacent nodes assigned to different participants. For each edge $\left(v_{i},v_{j}\right)$, where $\tau_{vp}\left(v_{i}\right)\neq \tau_{vp}\left(v_{j}\right)$, a send node and a receive node representing the inter-participant communication are added as follows:

- Add nodes $v_{send}$ and $v_{receive}$;
- Replace edge $\left(v_{i},v_{j}\right)$ by edges $\left(v_{i},v_{send}\right)$, $\left(v_{send},v_{receive}\right)$, and $\left(v_{receive},v_{j}\right)$;
- Set $\tau_{ew}\left(\left(v_{i},v_{send}\right)\right)$ = $\tau_{ew}\left(\left(v_{i},v_{j}\right)\right)$;
- Set $\tau_{vp}\left(v_{send}\right)$ = $\tau_{vp}\left(v_{i}\right)$ and $\tau_{vp}\left(v_{receive}\right)$=$\tau_{vp}\left(v_{j}\right)$;
- Add $v_{send}$ and $v_{receive}$ to the subgraphs of nodes $v_{i}$ and $v_{j}$, respectively;
- Set $\tau_{energy}\left(v_{send}\right)$=$\tau_{energyOut}\left(\tau_{vp}\left(v_{i}\right),\tau_{vp}\left(v_{j}\right)\right)$;
- Set $\tau_{energy}\left(v_{receive}\right)$=$\tau_{energyIn}\left(\tau_{vp}\left(v_{i}\right),\tau_{vp}\left(v_{j}\right)\right)$;
- Set $\tau_{rely}\left(v_{send}\right)$=$\tau_{relyOut}\left(\tau_{vp}\left(v_{i}\right),\tau_{vp}\left(v_{j}\right)\right)$;
- Set $\tau_{rely}\left(v_{receive}\right)$=$\tau_{relyIn}\left(\tau_{vp}\left(v_{i}\right),\tau_{vp}\left(v_{j}\right)\right)$;
- Set $\tau_{bv}\left(v_{send}\right)$ = $\tau_{bv}\left(v_{i}\right)$ and $\tau_{bv}\left(v_{receive}\right)$ = $\tau_{bv}\left(v_{j}\right)$.

If the new solution $s^{\prime }$ dominates the current solution *s*, the algorithm assigns $s^{\prime }$ to *s*. Otherwise, the algorithm also accepts the change between solutions with acceptance probability ProbAccept, as defined by Equation (10) [59].

(10) $$\begin{array}{cc} \; & ProbAccept(s^{\prime })=\\ \; & \min\left(1,\left(\exp\{\frac{-\Delta fr}{c}\}*\exp\{\frac{-\Delta fe}{c}\}\right)\right), \end{array}$$

where $\Delta fr=fr\left(s^{\prime }\right)-fr\left(s\right)$ and $\Delta fe=fe\left(s^{\prime }\right)-fe\left(s\right)$.

The control parameter *c* regulates how the current solution evolves in relation to its sensitivity when transitioning to a new solution [60]. Choosing an appropriate value for *c* ensures that new solutions with large increases in the objective values carry a low probability of acceptance, while those with smaller increases remain more likely to be accepted.

In each iteration, the value *c* is reduced by a constant factor σ, where σ is a positive constant smaller than but close to 1 (Equation (11)). As the value of *c* decreases, the algorithm becomes less likely to transition to dominated solutions.

(11) c=c∗σ

Algorithm 1 presents the pseudocode for our adapted SA algorithm. In this pseudocode, we have:

- *s* represents the current solution, initialised with $s_{start}$, the graph that represents the original BP;
- $s^{\prime }$ represents the newly generated neighbourhood solution;
- bestSol is the archive of potentially optimal solutions, initialised with solution $s_{start}$;
- *dominated* is an auxiliary boolean variable.

We define the stopping condition of the algorithm as c≈0 (line 2). Each iteration starts by generating a neighbour solution *s*′ from *s* (line 3) and defining the *dominated* boolean as false (line 4).

For each solution $s_{i}$ in the archive *bestSol* (line 5), we account for the scenario in which the solution $s^{\prime }$ has the same objective values as $s_{i}$ but is a different solution (line 6). In this scenario, we link *s*′ to $s_{i}$ (line 7).

Otherwise, if the solution *s*′ has at least one objective value different from $s_{i}$ and *s*′ dominates $s_{i}$, we remove the solution $s_{i}$ from the archive *bestSol* (lines 9–10). If $s_{i}$ dominates *s*′, *s*′ is set to *dominated* (lines 12–13).

If $s^{\prime }$ is dominated by one of the archived solutions, we update the current solution *s* to $s^{\prime }$ according to the probability computed by Equation (10) (lines 17–19). Otherwise, we add $s^{\prime }$ to the archive and update *s* to $s^{\prime }$ (lines 22–23).

As the transition between solutions is defined by a probabilistic function, our optimisation algorithm may get stuck in local optimal solutions. To avoid this, we implement a return-to-base strategy in which, at regular iteration intervals, the algorithm updates the current solution to an archived solution (line 25). At the end of each iteration, we update the *c* value (line 26).

In the next section, we detail how to use DECR with BPMN models.   
**Algorithm 1:** Pseudocode for the SA algorithm 1:Initialise $s=s_{start}$, bestSol={s}, dominated=false 2:**while** c≉0 **do** 3:    Generate a neighbour solution $s^{\prime }$ from *s* 4:    dominated=false 5:    **for** each solution $s_{i}\in bestSol$ **and not** dominated **do** 6:        **if** $fr\left(s_{i}\right)==fr\left(s^{\prime }\right)$ **and** $fe\left(s_{i}\right)==fe\left(s^{\prime }\right)$ **and** $s^{\prime }\neq s_{i}$ **then** 7:           Link $s^{\prime }$ to $s_{i}$ 8:        **else** 9:           **if** $s^{\prime }$ dominates $s_{i}$ **then**10:               Remove $s_{i}$ from bestSol11:           **end if**12:           **if** $s_{i}$ dominates $s^{\prime }$ **then**13:               dominated=true14:           **end if**15:        **end if**16:    **end for**17:    **if** dominated **then**18:        **if** $ProbAccept\left(s^{\prime }\right)\geq random\left(0,1\right)$ **then**19:           $s=s^{\prime }$20:        **end if**21:    **else**22:        Add $s^{\prime }$ to bestSol23:        $s=s^{\prime }$24:    **end if**25:    At regular iterations, execute the return-to-base strategy26:    c=c∗σ27:**end while**

## 4. Application of DECR to BPMN Models

BPMN is the de facto language adopted for modelling business processes [61]. Therefore, we elaborate on the application of the DECR methodology to BPMN models by defining the *decrBPMN* extension and describing how to generate the DECR hierarchical graph representation for a BPMN model.

### 4.1. The decrBPMN Extension

To enrich BPMN models with the information required for decentralisation decisions, we leveraged the *riskaBPMN* extension [62,63]. The *riskaBPMN* extension enriches BPMN models only with risk-related information. It adds risk-related information to the following BPMN elements: *Process*, *CategoryValue*, and *FlowNode* (*Activity*, *Event*, and *Gateway*). The *CategoryValue* is used to add risk-related information to a set of BPMN elements. In this way, reliability and business values, as well as risk computation, can be articulated at different levels of granularity.

The *riskaBPMN* extension defines three elements to store additional information: (1) the *RiskInformation* with the maximum acceptable risk value and the risk value; (2) the *ReliabilityInformation* with the reliability value; and (3) the *BusinessValueInformation* with the business value. It also defines the *Probability* element associated with the *SequenceFlow* BPMN element, which represents the transition probability of the conditional and loop patterns.

For the DECR methodology, we also need information on energy consumption values and mutable elements of BPMN. In addition, for mutable BPMN elements, the energy consumption and reliability depend on the participant executing them.

The optimisation algorithm in DECR can generate new communication flows between pools, which, in turn, lead to the emergence of new *send* and *receive* tasks. Consequently, it is necessary to know a priori information on the reliability and energy consumption values for each *send* and *receive* task, considering all possible combinations of source and target participants. Listing  A1 in Appendix A presents the XML Schema Definition (XSD) for the *decrBPMN* extension. It incorporates two additional components into the *riskaBPMN* extension: (1) the *EnergyInformation*, which includes energy consumption values (line 12), and (2) the *Mutable* element, which differentiates between mutable and fixed BPMN elements (line 14). In addition, each participant has assigned specific values to the *ReliabilityInformation* and *EnergyInformation* elements. Participants are defined using the *participantRef* attribute (lines 27 and 36).

The *OutgoingCommunication* element is employed within participant BPMN elements to define the reliability and energy consumption associated with *send* tasks pertaining to the message flows directed towards each of the other participants (line 15), while the *IncomingCommunication* element is utilised for *receive* tasks concerning the message flows originating from each of the other participants (line 16).

In the next subsection, we detail how we transform a BPMN model into a DECR graph.

### 4.2. From a BPMN Model to a DECR Graph Representation

The DECR methodology assumes that BPs and BPMN categories are structured as pattern blocks, including sequential, parallel, conditional, loop, and fault-tolerant.

Starting from the BPMN model, we create the graph G=(V,E,P,S,T,τ), as follows:

- Each BPMN *FlowNode* (*Task*, *Event*, and *Gateway*) is represented as a node of *V*.
- Each *Sequence Flow*, *Message Flow*, and *Data Association* is represented as an edge of *E*. The typing function $\tau_{ed}$ distinguishes control and data dependencies, as explained later in this subsection.
- *P* denotes the set of participants, which are graphically represented by *Pools* in the BPMN model.
- Each subgraph in *S* comprises the nodes that represent the BPMN elements with the same category value. For each category value cv within the BPMN model, we create the subgraph $S_{c}$ defined as $S_{c}=\left(V_{c},E_{c}\right)$, where $V_{c}=\left\{v\in V\right\}$ such that the category value of the BPMN element represented by *v* is cv, and $E_{c}=\{\left(u,v\right)\in E$ and $u,v\in V_{c}\}$. We consider that, by default, all nodes belong to category zero, thereby ensuring the composition of the root parent subgraph.
- Using $\tau_{vs}$, the nodes with more than one outgoing edge that represent an *Exclusive Gateway* are classified as xor-split nodes, while those nodes that represent a *Parallel Gateway* or a non-gateway BPMN element are classified as and-split nodes. The supported BPMN split gateways are the *Exclusive Gateway* and the *Parallel Gateway*.
- Using $\tau_{vj}$, the nodes that have more than one incoming edge and represent an *Exclusive Gateway* or a *Complex Gateway* are classified as xor-join nodes, while nodes that represent a *Parallel Gateway* or a non-gateway BPMN element are classified as and-join nodes. The supported BPMN join gateways are the *Exclusive Gateway*, *Complex Gateway*, and *Parallel Gateway*.
- Using $\tau_{vp}$, $\tau_{vp}\left(v\right)$ is the participant that executes the BPMN element represented by *v*, v∈V.
- Using $\tau_{vfm}$, v∈V is classified as *fixed* or *mutable* according to the value that the corresponding BPMN element has in the *Mutable* element of the extension.
- Using $\tau_{ed}$, an edge e∈E representing a *Sequence Flow* is classified as *control*, while the other edges are classified as *data*.
- Using $\tau_{ew}$, $\tau_{ew}\left(e\right)$, e∈E, is the value that the corresponding *Sequence Flow* has in the entity *Probability* of the extension.
- Using $\tau_{energy}$, $\tau_{rely}$, and $\tau_{bv}$, the corresponding values for the nodes and subgraphs are defined according to the values we have in the following elements of the extension: *EnergyInformation*, *ReliabilityInformation*, and *BusinessValueInformation*.
- Using $\tau_{energyOut}$, $\tau_{energyIn}$, $\tau_{relyIn}$, and $\tau_{relyOut}$, the energy consumption and reliability values are defined for sending and receiving messages, according to the values of the *OutgoingCommunication* and *IncomingCommunication* BPMN elements.

According to $\tau_{vs}$ and $\tau_{vj}$, the nodes that represent the *send* tasks are classified as and-split or none, while the nodes that represent the *receive* tasks are classified as and-join or none. In the following, we prune the edges between two nodes representing send and receive tasks (corresponding to empty parallel branches) and update the $\tau_{vs}$ and $\tau_{vj}$ of these nodes accordingly. These nodes are also pruned if their values of $\tau_{vs}$ and $\tau_{vj}$ are none. This pruning procedure aims at reducing the size of the graph and the size of the problem (see Section 3.4). In addition, the value of $\tau_{vfm}$ for these nodes is defined as *mutable* and the values of $\tau_{rely}$, $\tau_{bv}$, and $\tau_{energy}$ are set to the default values, which are 1, 0, and 0, respectively. The rationale for this option is that sensors have limited capacity for parallel execution; therefore, concurrent execution is considered to be translated into sequential code when the BPMN model is transcribed into code for IoT devices [64].

The next section focuses on the application and discussion of the DECR methodology.

## 5. Application and Discussion

This section first presents the application of the DECR methodology to the irrigation case study and examines the results of the corresponding sensitivity analysis. It then reports and discusses the outcomes of a computational study using pseudo-randomly generated BP models.

The purpose of the computational study is not to establish the superiority of the adapted simulated annealing algorithm over other multi-objective optimisation algorithms, but to assess DECR’s ability to generate decentralisation alternatives for BP models of increasing size.

### 5.1. BPMN Model of the Case Study

**Figure 2:**
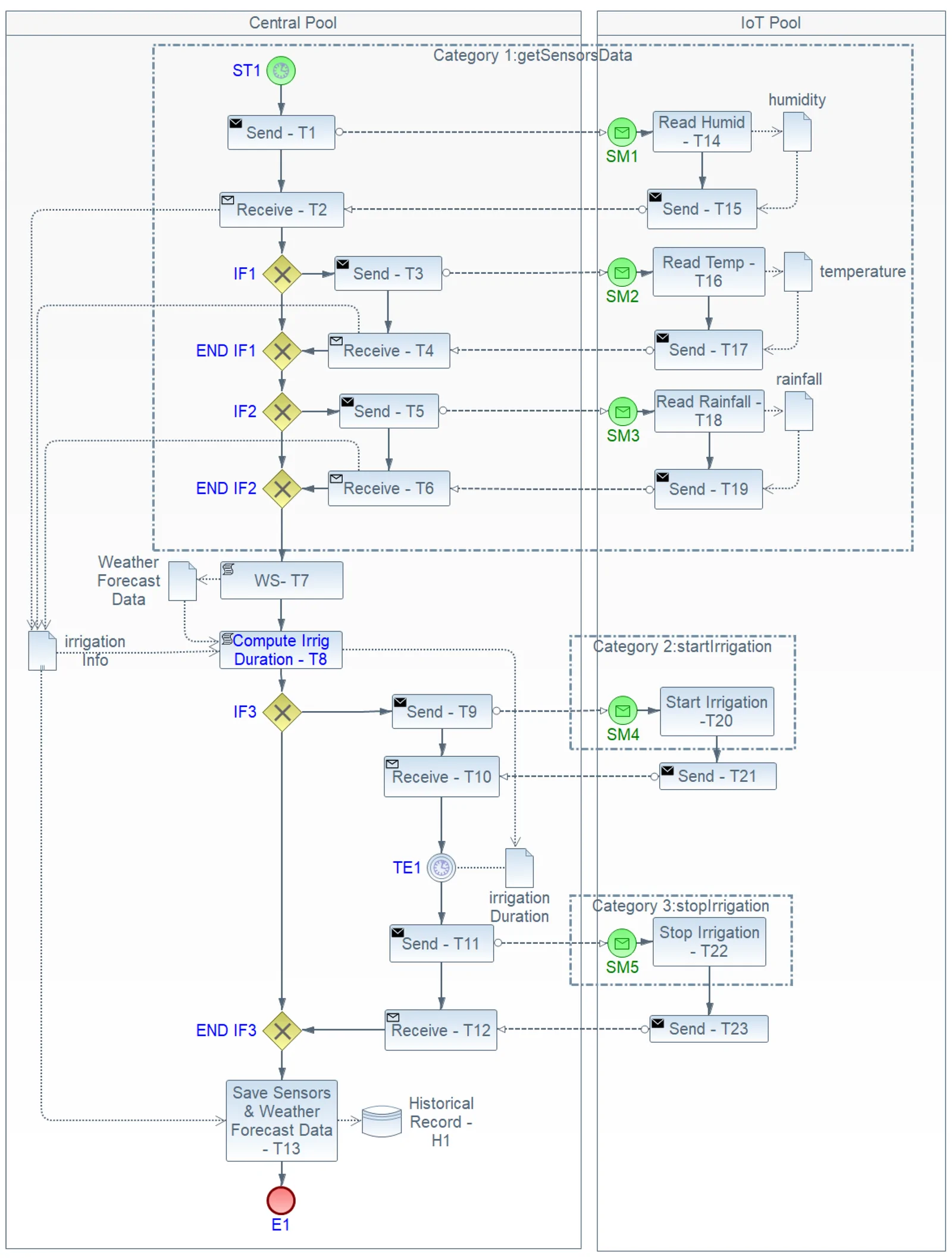
BPMN model of the irrigation process case study.

Figure 2 depicts a case study showcasing an irrigation system modelled using BPMN. The design of the case study draws inspiration from a real-world irrigation prototype developed in collaboration with the Lisbon city council [49,65]. This irrigation system aims to automate the watering process for public green areas, particularly when the workforce cannot manually supervise and manage irrigation.

The irrigation system employs IoT devices for sensing and actuation in the environment. Sensors measure environmental data on humidity, temperature, and rainfall. Actuator devices are responsible for opening and closing the irrigation electro-valves.

Our case study process includes a *Central Pool* and an *IoT Pool* that store the behaviour of the central system and IoT devices, respectively.

The process executes once a day, as defined by the *ST1* start timer event. Firstly, it gets the humidity value, sending a message to the *IoT Pool*, where the value is read and returned to the *Central Pool* (tasks *T1*, *SM1*, *T14*, *T15*, and *T2*). When needed, as defined by the gateway *IF1*, the process executes a similar sequence of tasks to get the temperature value (using tasks *T3*, *SM2*, *T16*, *T17*, and *T4*) and the rainfall value (using tasks *T5*, *SM3*, *T18*, *T19*, and *T6*).

These sensor values, together with the weather forecast information collected by task *T7*, are used to determine the irrigation duration (task *T8*). When irrigation is needed, considering the irrigation duration value (gateway *IF3*), the *Central Pool* communicates with the *IoT Pool* to open the valves and start irrigation (tasks *T9*, *SM4*, *T20*, *T21*, and *T10*).

The irrigation lasts for the duration of the *TE1* timer. Following this, the *Central Pool* initiates a request to the *IoT Pool*, signalling the closure of the valves and the cessation of irrigation (tasks *T11*, *SM5*, *T22*, *T23*, and *T12*).

Finally, task *T13* saves the sensor and weather forecast data from the current execution of the process.

In addition, to represent the irrigation system, the BPMN model also includes groups of BPMN elements, designated in the BPMN terminology as categories. These categories are aptly named according to their purposes, such as *Category 1: getSensorsData*, *Category 2: startIrrigation*, and *Category 3: stopIrrigation*.

We also identify the mutable BPMN elements, distinguishing them by colouring their labels in blue. These elements are *ST1*, *IF1*, *END IF1*, *IF2*, *END IF2*, *T8*, *IF3*, *TE1*, *END IF3*, and *E1*.

Listing A2 in Appendix A presents an extract of the XML of this BPMN model, enriched with information related to risk (reliability and business value) and energy consumption, using the *decrBPMN* extension. In this example, we present the information about *Category 1*, namely the business value (line 3). We also have the two BPMN participants: the *Central Pool* (line 8) and the *IoT Pool* (line 14). Within these BPMN elements, we define the reliability and energy consumption values for the send tasks that communicate from the *Central Pool* to the *IoT Pool* (line 10) and from the *IoT Pool* to the *Central Pool* (line 16). Similarly, we define the reliability and energy consumption values for the receive tasks that ensure communication from the *IoT Pool* to the *Central Pool* (line 11) and from the *Central Pool* to the *IoT Pool* (line 17).

Moreover, with *ST1*, we exemplify how BPMN elements can be enriched with information related to risk and energy consumption, namely the energy consumption value for participant 2 (line 22) and the mutable information (line 23).

### 5.2. DECR Methodology Application to the Case Study

To apply the DECR methodology to the irrigation case study, we first enrich the BPMN model with risk-related and energy consumption information, using the values presented in Table 2. This table also includes the references that justify each value.

The branches of the *IF1* and *IF2* gateways, which include *T16* and *T18*, respectively, have probabilities of 0.6 and 0.7, respectively, indicating that additional environmental data may be required to determine whether irrigation is necessary.

The branch of the *IF3* gateway that includes *T20* has a probability of 0.7. This probability reflects the need to water the crop [18].

The reliability value of the *IncomingCommunication* element of the *Central Pool* is 0.992 [69]. For the *IoT Pool*, the energy consumption of the *OutgoingCommunication* element is 9.3357 [70], while the reliability value and the energy consumption of the *IncomingCommunication* element are 0.992 [69] and 1.93248 [70], respectively. The remaining values are set to their respective default values.

The graph representing the BP case study has 10 mutable tasks and two participants. Therefore, the size of the optimisation problem is $2^{10}$. The control parameter *c* is set to $10^{6}$, the reduction factor σ to 0.9997, and the return-to-base strategy is applied every $10^{3}$ iterations.

Since the optimisation algorithm is probabilistic, its results may vary between runs. To reduce this variability and increase the likelihood of finding solutions near the global optimum, the algorithm is executed ten times.

In this context, the algorithm yielded two solutions. In Table 3, we present the risk and energy consumption values and the number of communication tasks for the initial model and for the two solutions.

In both solutions, the nodes representing *IF1*, *END IF1*, *IF2*, and *END IF2* are moved to the *IoT Pool*, reducing the number of communication tasks, the energy consumption, and the risk values. The differences between the two potentially optimal solutions concern the participant who executes *ST1*. When *ST1* is executed by the *Central Pool*, the energy consumption value decreases; when it is executed by the *IoT Pool*, the risk value decreases due to an increase in the reliability value, as one pair of communication tasks becomes unnecessary.

The limited size of the irrigation case study, comprising 10 mutable nodes and two participants, allowed us to exhaustively enumerate all 1024 feasible assignments. This enumeration was used as a correctness check for this specific problem instance, confirming that the two solutions identified by the adapted simulated annealing algorithm are Pareto-efficient. The results also illustrate the trade-off between energy consumption and business risk in the considered irrigation process. This instance-specific verification is not intended as a general performance benchmark.

### 5.3. Sensitivity Analysis

To evaluate the stability of the obtained solutions with respect to parameter estimation uncertainty, we conducted a sensitivity analysis. The following input parameters were identified as relevant:

- Reliability of communications;
- Reliability of IoT devices;
- Business values associated with *Category 1* and *Category 2*;
- Energy consumption of timers;
- Energy consumption of communications;
- Energy consumption of IoT devices.

The values of the input parameters vary by −20%, −10%, +10% and +20%. Reliability values are limited to 0.99999 when needed.

The tables in Appendix B present the results. This sensitivity analysis shows that the solutions are stable under the considered variations. Indeed, in all tested scenarios, the number of optimal solutions and the corresponding models remain unchanged.

It should be noted that the risk values computed by DECR depend on the quality and uncertainty of the underlying reliability and business value estimates. In particular, when they are derived from, for instance, limited historical data or expert judgement, estimation errors may affect the calculated risk values and, consequently, the generated decentralisation alternatives. Although this sensitivity analysis indicates that the identified solutions remain stable under the parameter variations considered in the irrigation case study, this result cannot be generalised. Therefore, the generated Pareto front should be interpreted in light of the assumptions and uncertainty associated with the input parameters. When these estimates are uncertain, stakeholders may assess the robustness of the alternatives by repeating the optimisation under plausible parameter variations before making a final decentralisation decision.

### 5.4. Computational Study

For this computational study, we developed a generator of pseudo-random BPs that creates event-driven or timer-event BPs, which align with the IoT-aware BP types in our study. We configured the generator with the following parameters:

- Number of participants: 3;
- Number of mutable nodes: 10, 20, 30, or 40;
- Reliability of fixed nodes of participant 2: 0.88–0.98;
- Energy consumption of fixed nodes of participant 2: 0.2–0.7;
- Reliability of fixed nodes of participant 3: 0.85–0.88;
- Energy consumption of fixed nodes of participant 3: 25–27;
- Business value of categories: 22,000–28,000;
- Reliability of communication nodes between participants: 0.955–0.995;
- Energy consumption of send nodes between participants: 8–2;
- Energy consumption of receive nodes between participants: 1–3.

The reliability and energy consumption values for the fixed nodes of participants 2 and 3 are based on the sensor and actuator values of our case study.

The business values for the categories are based on those of Category1 and Category2 (Table 2). As the business value of Category3 is significantly lower, we decided to close the gap in the possible business values.

Finally, the reliability of communications is based on the varying heights and distances from LoRa antennas [69], and their energy consumption depends on the values in the case study.

As we increased the number of participants for this computational study, we set the reduction factor σ to 0.9998. The other algorithm parameters were set to the same values as in the case study.

We generated four sets of BPs, each consisting of 30 BPs with 10, 20, 30, or 40 mutable nodes, using the aforementioned values. Table 4 presents, for each set, the percentage of BPs generated with two and three or more solutions and the highest number of solutions found for a single BP.

The results show that multiple potentially Pareto-efficient alternatives are frequently identified across all problem sizes. Specifically, 66.7% of the BPs with 10 mutable nodes and 60% of those with 20 mutable nodes yielded at least two solutions. For BPs with 30 and 40 mutable nodes, the corresponding proportions were 63.4% and 80.0%, respectively. Moreover, the proportion of BPs yielding three or more solutions increased from 20% for 10 and 23.3% for 20 mutable nodes to 36.7% for 30 mutable nodes and 53.3% for 40 mutable nodes. The maximum number of solutions found for a single BP also reached 14 for the set with 30 mutable nodes. These results show that the decentralisation problem often admits multiple non-dominated alternatives and that richer Pareto sets tend to emerge in larger instances. This supports the use of a multi-objective optimisation approach, since providing process designers with multiple alternatives makes the trade-off between business risk and energy consumption explicit rather than prescribing a single decentralisation solution.

In Table 5, we present, for the generated BP with the highest number of solutions found, the risk-related and energy consumption values for the initial model and the potentially optimal solutions, along with the number of linked solutions (see Section 3.5). For this BP, the algorithm found 14 solutions in the objective space, and two of them yield two alternatives in the solution space. The latter correspond to alternative BP models for the same objective values that can be assessed considering other criteria relevant for the BP design.

Regarding computational complexity, the number of SA iterations is determined by the initial control parameter *c* and the reduction factor σ. At each iteration, a neighbour solution is generated, and its risk and energy consumption values are evaluated. For the sequential, parallel, conditional, and loop patterns, the corresponding reduction operations are at most linear in the number of nodes of the pattern block. The main exception is the reliability computation for a fault-tolerant pattern with *b* branches. This computation considers all $2^{b}$ possible combinations of successful and failed branches and performs the number of operations needed to reduce the branches for each combination, resulting in exponential complexity. In addition, each candidate solution is compared with the solutions currently stored in the archive of non-dominated solutions. This contributes to a computational cost bounded by the number of solutions in the archive.

The results of Table 4 also show that the average execution time increases with the number of mutable nodes, from 4963 ms for BPs with 10 mutable nodes to 63,424 ms for those with 40 mutable nodes. Since in this computational study all experiments execute the same number of SA iterations (138,143), these numbers reflect the increase in the average computational cost per iteration as the BP size grows, as well as the increase in the size of the non-dominated solutions archive. The increasing standard deviation of execution time is consistent with variability among the BPs in their control-flow structure, number of additional communication nodes, and total number of nodes. For instance, the number of generated fixed nodes for BPs with 40 mutable nodes varies from 120 to 319, and the minimum and maximum execution times were obtained for the BPs with the minimum and maximum number of nodes, respectively.

To analyse the empirical stabilisation of the archive of the algorithm, we also recorded the stabilising iteration, defined as the iteration after which the set of potentially non-dominated solutions remains unchanged until the end of the run. Nevertheless, the archive stabilisation does not guarantee convergence to the true Pareto front. The average stabilising iteration increases with problem size, from 36,165 iterations for 10 mutable nodes to 79,699 iterations for 40 mutable nodes. In addition, for all four problem sizes, the archive stabilises, on average, well before the termination of the algorithm. This observation aligns with our purpose, which is to obtain a good approximation of the Pareto front rather than prioritise computational speed.

In the following section, we present our final remarks on the work and discuss future improvements.

## 6. Conclusions

This paper addresses the design-time decentralisation of IoT-aware business processes by jointly considering energy consumption and business risk, two critical criteria in IoT environments.

While existing proposals primarily focus on operational criteria, such as energy consumption, communication overhead, or reliability, they provide limited support for evaluating the business consequences of decentralisation decisions. To overcome this limitation, we propose DECR, a decision-support methodology that explicitly integrates risk as the expected impact of failures on the business, combining reliability and business value. This supports decisions aligned with organisational objectives. Moreover, DECR provides potentially Pareto-efficient alternatives that stakeholders can use to analyse the trade-off between business risk and energy consumption.

The DECR methodology includes a modelling approach for IoT-aware BPs that enriches models with the information required for decentralisation decisions. In addition, DECR formulates decentralisation as a bi-objective generalised assignment problem and solves it using an adapted bi-objective simulated annealing algorithm that generates potentially Pareto-efficient decentralisation alternatives. The application of DECR to the irrigation case study demonstrated that decentralisation decisions involve a genuine trade-off between energy consumption and business risk. The multiple Pareto-efficient alternatives showed that energy consumption and business risk are inherently conflicting criteria in IoT environments, and that ignoring one of them may lead to suboptimal or misaligned decentralisation decisions. The sensitivity analysis further indicated that the identified alternatives remain stable under variations in the main input parameters. In addition, the computational study using pseudo-randomly generated business processes showed that multiple potentially Pareto-efficient alternatives frequently emerge, reinforcing the importance of supporting stakeholders with a set of decision alternatives rather than a single optimised solution.

From a decision-support perspective, the main contribution of DECR is the explicit exposure of the trade-offs between competing decentralisation objectives. Instead of prescribing a single solution, DECR enables stakeholders to analyse alternative decentralisation models based on their operational constraints, business priorities, and risk acceptance criteria. This capability is particularly relevant in IoT environments, where the consequences of failures may differ significantly depending on the business criticality of the affected process elements.

This methodology can also be applied in other contexts where risk is unavoidable, while considering alternative cost dimensions, such as monetary costs for human resources or cloud resource usage, latency, or carbon footprint.

Although DECR is not conceptually restricted to the irrigation domain, its evaluation has so far been limited to a real-world irrigation case study and a computational study using pseudo-randomly generated business processes. Therefore, its applicability to other domains remains to be experimentally validated.

Future work will focus on extending the approach to support less structured business process models and on evaluating its applicability in additional domains, including healthcare, industrial IoT, and cloud-edge computing environments.

To this end, the DECR methodology has to be extended to consider participants’ capacity limitations. To address this, the model has to include information on participants’ capacities and the resources needed by each node. In addition, the optimisation algorithm has to consider those constraints to generate only feasible alternatives.

Furthermore, future research may also investigate interactive decision-support mechanisms to assist stakeholders in selecting solutions from the generated Pareto frontier.

In summary, this work advances the state of the art by explicitly integrating business risk and energy consumption into the decentralisation of IoT-aware BPs, enabling more informed, business-aligned design decisions in distributed IoT environments.

## Figures and Tables

**Table 1:** Formulas to compute risk-related components and energy consumption for different BP pattern blocks.

Pattern Name	Pattern Design	Formulas
Sequential	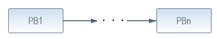	$\begin{array}{cc} BV(Seq\left[PB_{1},\ldots ,PB_{n}\right]) & =\max(BV\left(PB_{1}\right),\ldots ,BV\left(PB_{n}\right))\\ Rely(Seq\left[PB_{1},\ldots ,PB_{n}\right]) & =\prod_{i=1}^{n}Rely\left(PB_{i}\right)\\ Risk(Seq\left[PB_{1},\ldots ,PB_{n}\right]) & =\max(Risk\left(PB_{1}\right),\ldots ,Risk\left(PB_{n}\right))\\ Energy(Seq\left[PB_{1},\ldots ,PB_{n}\right]) & =\sum_{i=1}^{n}Energy\left(PB_{i}\right) \end{array}$
Parallel	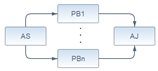	$\begin{array}{cc} BV(Par\left[PB_{1},\ldots ,PB_{n}\right]) & =\max(BV\left(PB_{1}\right),\ldots ,BV\left(PB_{n}\right))\\ Rely(Par\left[PB_{1},\ldots ,PB_{n}\right]) & =\prod_{i=1}^{n}Rely\left(PB_{i}\right)\\ Risk(Par\left[PB_{1},\ldots ,PB_{n}\right]) & =\max(Risk\left(PB_{1}\right),\ldots ,Risk\left(PB_{n}\right))\\ Energy(Par\left[PB_{1},\ldots ,PB_{n}\right]) & =\sum_{i=1}^{n}Energy\left(PB_{i}\right) \end{array}$
Conditional	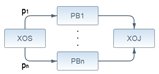	$\begin{array}{cc} BV(Cond\left[PB_{1},\ldots ,PB_{n}\right]) & =\max(p_{1}BV\left(PB_{1}\right),\ldots ,p_{n}BV\left(PB_{n}\right))\\ Rely(Cond\left[PB_{1},\ldots ,PB_{n}\right]) & =\sum_{i=1}^{n}p_{i}Rely\left(PB_{i}\right)\\ Risk(Cond\left[PB_{1},\ldots ,PB_{n}\right]) & =\max(p_{1}Risk\left(PB_{1}\right),\ldots ,p_{n}Risk\left(PB_{n}\right))\\ Energy(Cond\left[PB_{1},\ldots ,PB_{n}\right]) & =\sum_{i=1}^{n}p_{i}Energy\left(PB_{i}\right) \end{array}$
Loop	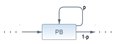	$\begin{array}{cc} BV(Loop[PB]) & =BV(PB)\\ Rely(Loop[PB]) & =\frac{\left(1-p)\;Rely(PB\right)}{1-p\;Rely(PB)}\\ Risk(Loop[PB]) & =Risk(PB)\\ Energy(Loop[PB]) & =\frac{Energy(PB)}{1-p} \end{array}$
Fault-Tolerant	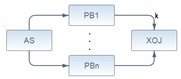	$\begin{array}{cc} BV(FT\left[PB_{1},\ldots ,PB_{n}\right]) & =\max(BV\left(PB_{1}\right),\ldots ,BV\left(PB_{n}\right))\\ Rely(FT\left[PB_{1},\ldots ,PB_{n}\right]) & =\sum_{I_{1}=0,1}\cdots \sum_{I_{n}=0,1}[\;\phi \left(\sum_{i=1}^{n}I_{i}-k\right)\\ & \;\times \prod_{i=1}^{n}\left(1-I_{i}+\left(2I_{i}-1\right)\;Rely\left(PB_{i}\right)\right)],\\ & \;where\;\phi (x)=1,if\;x\geq 0;\;0,\;otherwise\\ Risk(FT\left[PB_{1},\ldots ,PB_{n}\right]) & =\max(Risk\left(PB_{1}\right),\ldots ,Risk\left(PB_{n}\right))\\ Energy(FT\left[PB_{1},\ldots ,PB_{n}\right]) & =\sum_{i=1}^{n}Energy\left(PB_{i}\right) \end{array}$

**Table 2:** Risk-related information and energy consumption of the case study BP.

BP Elements	IoT Pool Reliability	Business Value	IoT Pool Energy Consumption
Category1, T7, T8, IF3, T9, Category2		25,730 [18]	
T11, Category3		78 [18]	
TE1			1.903707 [66]
ST1			27.393 [66,67,68]
SM1, SM2, SM3, SM4, SM5	0.992 [69]		1.93248 [70]
T2, T4, T6			
T10		78 [18]	
T12			
T15, T17, T19, T23			9.3357 [70]
T21		78 [18]	
T14	0.9707 [71,72]		0.1932 [73,74]
T16	0.9888 [71,72]		
T18	0.8886 [71,72]		0.638 [73,74]
T20	0.87954 [71,72]		33 [75]
T22	0.85674 [71,72]		

**Table 3:** Results of the case study BP.

Solution ID	Risk Value	Energy Consumption	# Comm Tasks
Initial Model	3674.736	88.65	20
Solution 1	3039.267	**67.44**	8
Solution 2	**2856.277**	92.91	6

**Table 4:** Results of the pseudo-randomly generated BPs.

# Mutable Nodes	Range of # Fixed Nodes	Two Solutions (%)	Three or More Solutions (%)	Max Number Solutions	Average Execution Time (ms)	Standard Deviation Execution Time (ms)	Average Stabilising Iteration
10	[11; 26]	46.7%	20.0%	7	4963	918	36,165
20	[11; 45]	36.7%	23.3%	7	11,380	1245	59,920
30	[34; 103]	26.7%	36.7%	14	24,166	3589	63,086
40	[120; 319]	26.7%	53.3%	12	63,424	15,936	79,699

**Table 5:** Results of a pseudo-randomly generated BP.

Solution ID	Risk Value	Energy Consumption Value	# Linked Solutions
Initial Model	9997.059	343.557	
Solution 1	9707.826	**332.162**	1
Solution 2	9658.860	335.058	1
Solution 3	9531.310	353.389	1
Solution 4	9531.308	368.342	1
Solution 5	9470.054	370.077	1
Solution 6	9413.319	373.218	1
Solution 7	9393.496	374.144	1
Solution 8	9112.236	374.708	1
Solution 9	9087.339	390.407	1
Solution 10	9043.676	391.481	1
Solution 11	8913.119	396.072	2
Solution 12	8836.525	411.441	1
Solution 13	8773.851	441.390	1
Solution 14	**8646.329**	444.947	2

## Data Availability

The data that support the findings of this study are available from the authors in the *GitHub* repository https://github.com/PedroBandeirasCardoso/DECR (accessed on 2 August 2026).

## Appendix A. XSD and XML Snippet of the *decr*BPMN Extension

**Listing A1.** XSD schema for decrBPMN.

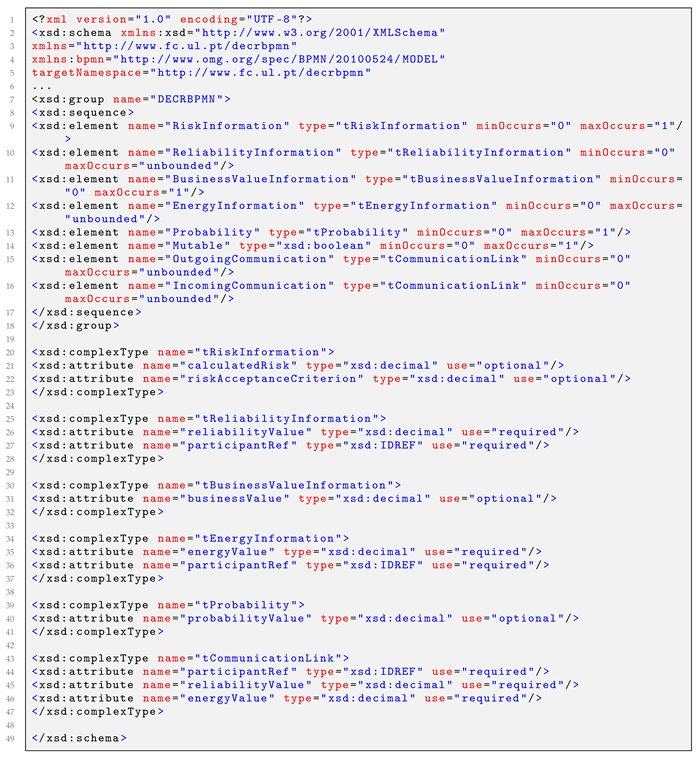

**Listing A2.** XML snippets exemplifying the application of the decrBPMN extension to add risk-related and energy information.

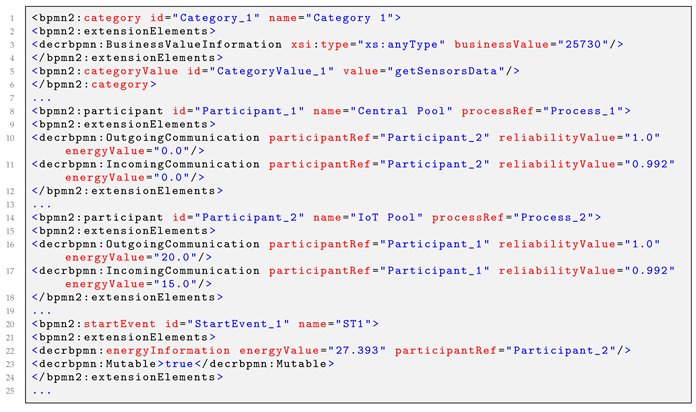

## Appendix B. Results of the Sensitivity Analysis

**Table A1:** Sensitivity assessment for communication reliability.

Solution ID	Comms Relys −20%	Comms Relys −10%	Comms Relys Near 1
Initial Solution	22,186.513 88.65	16,870.72 88.65	3268.44 88.65
Solution 1	14,112.345 67.44	9188.456 67.44	2970.486 67.44
Solution 2	7431.022 92.91	5143.65 92.91	2879.151 92.91

**Table A2:** Sensitivity assessment for the IoT device reliability.

Solution ID	IoT Rely −20%	IoT Rely −10%	IoT Rely Near 1
Initial Solution	12,266.997 88.65	8311.242 88.65	3120.898 88.65
Solution 1	119,02.562 67.44	7823.526 67.44	2467.59 67.44
Solution 2	11,791.05 92.91	7679.119 92.91	2279.989 92.91

**Table A3:** Sensitivity assessment for the business values of *Category 1* and *Category 2*.

Solution ID	Cat1 & Cat2 BV −20%	Cat1 & Cat2 BV −10%	Cat1 & Cat2 BV +10%	Cat1 & Cat2 BV +20%
Initial Solution	2939.789 88.65	3307.263 88.65	4042.21 88.65	4409.684 88.65
Solution 1	2431.414 67.44	2735.341 67.44	3343.194 67.44	3647.121 67.44
Solution 2	2285.022 92.91	2570.65 92.91	3141.905 92.91	3427.533 92.91

**Table A4:** Sensitivity assessment for the timers’ energy consumption.

Solution ID	Timers EC −20%	Timers EC −10%	Timers EC +10%	Timers EC +20%
Initial Solution	3674.736 88.65	3674.736 88.65	3674.736 88.65	3674.736 88.65
Solution 1	3039.267 67.18	3039.267 67.31	3039.267 67.57	3039.267 67.71
Solution 2	2856.277 87.16	2856.277 90.03	2856.277 95.77	2856.277 98.65

**Table A5:** Sensitivity assessment for communication energy consumption.

Solution ID	Comms EC −20%	Comms EC −10%	Comms EC +10%	Comms EC +20%
Initial Solution	3674.736 80.31	3674.736 84.47	3674.736 92.82	3674.736 96.99
Solution 1	3039.267 63.61	3039.267 65.53	3039.267 69.36	3039.267 71.28
Solution 2	2856.277 89.46	2856.277 91.18	2856.277 94.63	2856.277 96.35

**Table A6:** Sensitivity assessment for the IoT devices’ energy consumption.

Solution ID	IoT EC −20%	IoT EC −10%	IoT EC +10%	IoT EC +20%
Initial Solution	3674.736 79.25	3674.736 83.95	3674.736 93.34	3674.736 98.04
Solution 1	3039.267 58.05	3039.267 62.75	3039.267 72.14	3039.267 76.84
Solution 2	2856.277 83.51	2856.277 88.21	2856.277 97.6	2856.277 102.3
